# SARS-CoV-2 Causes a Systemically Multiple Organs Damages and Dissemination in Hamsters

**DOI:** 10.3389/fmicb.2020.618891

**Published:** 2021-01-12

**Authors:** Zhiqi Song, Linlin Bao, Pin Yu, Feifei Qi, Shuran Gong, Jie Wang, Binbin Zhao, Mingya Liu, Yunlin Han, Wei Deng, Jiangning Liu, Qiang Wei, Jing Xue, Wenjie Zhao, Chuan Qin

**Affiliations:** NHC Key Laboratory of Human Disease Comparative Medicine, Beijing Key Laboratory for Animal Models of Emerging and Remerging Infectious Diseases, Institute of Laboratory Animal Science, Chinese Academy of Medical Sciences and Comparative Medicine Center, Peking Union Medical College, Beijing, China

**Keywords:** SARS-CoV-2, COVID-19, Syrian hamsters, pathological changes, systemic damages, adrenal gland, alimentary system, animal model

## Abstract

Severe acute respiratory syndrome coronavirus-2 (SARS-CoV-2) has spread across the world and impacted global healthcare systems. For clinical patients, COVID-19 not only induces pulmonary lesions but also affects extrapulmonary organs. An ideal animal model that mimics COVID-19 in humans in terms of the induced systematic lesions is urgently needed. Here, we report that Syrian hamster is highly permissive to SARS-CoV-2 and exhibit diffuse alveolar damage and induced extrapulmonary multi-organs damage, including spleen, lymph nodes, different segments of alimentary tract, kidney, adrenal gland, ovary, vesicular gland and prostate damage, at 3–7 days post inoculation (dpi), based on qRT-PCR, *in situ* hybridization and immunohistochemistry detection. Notably, the adrenal gland is a novel target organ, with abundant viral RNA and antigen expression detected, accompanied by focal to diffuse inflammation. Additionally, viral RNA was also detected in the corpus luteum of the ovary, vesicular gland and prostate. Focal lesions in liver, gallbladder, myocardium, and lymph nodes were still present at 18 dpi, suggesting potential damage after disease. Our findings illustrate systemic histological observations and the viral RNA and antigen distribution in infected hamsters during disease and convalescence to recapitulate those observed in humans with COVID-19, providing helpful data to the pathophysiologic characterization of SARS-CoV-2-induced systemic disease and the development of effective treatment strategies.

## Introduction

The golden Syrian hamster (*Mesocricetus auratus*) is a commonly used experimental animal model that has been recorded to support the replication of SARS-CoV (Roberts et al., 2005) and SARS-CoV-2 (Sia et al., 2020) but not MERS-CoV (de Wit et al., 2013), which capitalizes on the dipeptidyl peptidase-4 (DPP4) protein as the central receptor for viral entry. Syrian hamster have been put to use as animal models for other respiratory system related viruses (Wang et al., 2019), including SARS-CoV, influenza virus, and adenovirus (Hammer and McPhee, 2014; Song et al., 2019; Wang et al., 2019). *In silico* comparison of the angiotensin-converting enzyme 2 (ACE2) sequence of humans known to interact with the SARS-CoV-2 spike glycoprotein receptor-binding domain (RBD) with that of hamsters (Chan et al., 2020) implied that Syrian hamsters might be susceptible to SARS-CoV-2 infection. Recently, the hamster has been widely used as a small animal for building up the model of SARS-CoV-2 infection and developing countermeasure. After experimental intranasal infection, Syrian hamsters exhibit mild to moderate disease with progressive body weight loss starting very early after infection [1–2 days post inoculation (dpi)]. All animals inoculated by different SARS-CoV-2 isolates and with different groups consistently show respiratory symptoms, including labored breathing (Boudewijns et al., 2020). Additional features of morbidity include lethargy, ruffled fur and hunched posture (Chan et al., 2020). Two weeks after infection, hamsters typically recover. SARS-CoV-2 disease in hamsters is related to high levels of virus replication and pathological changes in respiratory tract (Chan et al., 2020).

Clinically, persistent symptoms in patients during and after recovery from COVID-19, such as diarrhea, hepatic damage, renal failure, fatigue, dyspnea, joint pain, chest pain (Carfì et al., 2020) and neurologic manifestations (Mao et al., 2020), are found. However, at present, there are many unknowns, and few reports have observed extrapulmonary and systemic lesions in animal models to mimic the potential pathogenic process in real patients with COVID-19. On the other hand, histopathological findings have only been collected in fatal cases. The critical period for disease development and convalescent pathology is still unclear. As a small animal model for SARS-CoV-2 infection, Syrian hamsters display efficient SARS-CoV-2 replication in their lungs and exhibit severe pathological changes in the lungs similar to generally reported imaging features of patients with COVID-19. However, previous reports demonstrated that among non-respiratory tract tissues, only intestinal tissue demonstrated viral antigen expression in combination with severe epithelial cell necrosis, intestinal villi damage and deformation, and increased *lamina propria* mononuclear cell infiltration (Boudewijns et al., 2020; Imai et al., 2020). At the whole-body level, understanding the relationship between extrapulmonary organ injury and COVID-19 is crucial but still not clear.

Here, Syrian hamsters were challenged with SARS-CoV-2 and observed for 37 days, and the pathological characteristics of the infected hamsters during disease and recovery were recorded to provide systemic pathological data for understanding the pathogenesis of COVID-19 in patients. Remarkably, we found that the adrenal gland is an important target organ of SARS-CoV-2, based on observation by hematoxylin and eosin (H&E) staining, examination of viral RNA by *in situ* hybridization (ISH), and detection of viral antigen by immunohistochemistry (IHC). Viral RNA was also detected in the corpus luteum of the ovary, vesicular gland and prostate. Overall, hamsters provide a commercially available small animal model in which the clinical, virological, and histopathological hallmarks of SARS-CoV-2 infection appear to recapitulate those observed in humans with COVID-19.

## Materials and Methods

### Ethics Statement

At the Institute of Laboratory Animal Science (ILAS), the animal biosafety level 3 (ABSL3) facility was used to accomplish all the research with Syrian hamsters (male and female, aged 12 weeks old). All experiments were implemented according to the Animal Welfare Act and other regulations associated with animals and experiments. The Institutional Animal Care and Use Committee (IACUC) of the ILAS, Peking Union Medical College, evaluated and gave permission to all the protocols in these studies, including animal experiments (LJN20004).

### Cells and Viruses

The SARS-CoV-2 virus (accession number is MT093631.2, SARS-CoV-2/WH-09/human/2020/CHN) (Deng et al., 2020a) was put to use in this research and seeded in Vero E6 cells incubated in Dulbecco’s modified Eagle’s medium (Invitrogen, Carlsbad, United States) supplemented with 10% fetal bovine serum (Gibco, Grand Island, United States) and incubated at 37°C and five percent carbon dioxide.

### Syrian Hamsters Experiments

To monitor viral replication and histopathological changes, eighteen hamsters were inoculated intranasally with SARS-CoV-2 stock virus at 10^6^ 50% tissue culture infectious dose (TCID_50_) per mL (0.2 mL per animal) used as SARS-CoV-2 challenge group. Three hamsters were inoculated intranasally with an equal volume of PBS were used as a mock control group. The infected and mock-infection hamsters were continuously observed to record clinical symptoms, body weight, responses to external stimuli, and death. Throat swabs were collected and incubated in 1 mL of DMEM containing 50 μg/mL streptomycin and 50 U/mL penicillin on 0, 1, 3, 5, and 7 dpi. The main organs were collected in the SARS-CoV-2 challenge group at 1, 3, 5, 7, 18, and 37 dpi (*N* = 3/group) and in the mock control group at 7 dpi (*N* = 3).

### RNA Extraction and Quantitative Real-Time Polymerase Chain Reaction (qRT-PCR)

Utilizing the RNeasy Mini Kit (Qiagen, Hilden, Germany), total RNA was extracted from all the collected tissues and organs. According to the manufacturer’s explanations of the PrimerScript RT Reagent Kit (TaKaRa, Japan), reverse transcription was completed (Deng et al., 2020a; Gao et al., 2020). Putting to use the PowerUp SYBR Green Master Mix Kit (Applied Biosystems, United States), qRT-PCR was implemented, and then all the samples were prepared in duplicate followed the cycling protocol: 50°C for two min; 95°C for two min; 40 cycles of 95°C for 15 s, 60°C for thirty s, and 95°C for 15 s; 60°C for 1 min; and 95°C for 45 s. The primer sequences directed at qRT-PCR targeted the envelope protein (E) gene of SARS-CoV-2 and were as follows: forward: 5′-TCGTTTCGGAAGAGACAGGT-3′, reverse: 5′-GCGCAGTAAGGATGGCTAGT-3′. Using an ABI 3730 DNA sequencer (Applied Biosystems, CA, United States), the PCR products were confirmed by sequencing with the dideoxy method. During the sequencing course, amplification was completed with specific primers. The obtainable sequencing reads were linked with DNAMAN. In the DNAStar software package, the results were compared with the MEGALIGN module. The sequences of the primers prepared for this program were available upon request.

### Homogenate Supernatant Preparation

For preparing the tissue homogenates (1 g/1 mL), an electric homogenizer was performed by maintaining in 1 mL of DMEM for 2.5 min. At 825 × *g* at 4°C for 10 min, the homogenates were centrifuged (Bao et al., 2020b; Deng et al., 2020a).

### TCID_50_ Assay

The TCID_50_ assay was performed as following (Bao et al., 2020a; Deng et al., 2020b), briefly, to mensurate the SARS-CoV-2 titers, 10-fold serial dilutions of the virus were applied to seed Vero E6 cell monolayers in DMEM including two percent fetal bovine serum, which were maintained in 37°C for 4 days. The cytopathic effect (CPE) was recorded. Via the Reed and Muench method, the TCID_50_ values were calculated.

### Hematoxylin and Eosin (HE) Staining

A ten percent buffered formalin solution was applied to fix all the collected tissues and organs, and paraffin sections (3–4 μm in thickness) were applied following the routine operating procedure. All the tissue and organs sections were stained with HE. The pathological findings in diverse tissues and organs were carefully observed using an Olympus microscope.

### Immunohistochemistry (IHC)

A 10 percent buffered formalin solution was applied to fix all the collected tissues and organs, and paraffin sections (3–4 μm in thickness) were completed routinely as described in a previous report (Deng et al., 2020a; Yu et al., 2020). Briefly, reagent (Boster, AR0022) from an antigen retrieval kit was prepared to treat the sections at 37°C for one min. A three percent solution of H_2_O_2_ in methanol was used to quench endogenous peroxidases for 10 min. With anti-SARS spike glycoprotein mouse monoclonal antibody (Abcam, ab273433, 1:1000), the slices were maintained in 4°C overnight, synaptophsin rabbit monoclonal antibody (Abcam, ab32127, 1:1000), CD31 rabbit polyclonal antibody (Abcam, ab28364, 1:100), or CD19 rabbit monoclonal antibody (Abcam, ab245235,1:1000) after blocking in one percent normal goat serum. The goat anti-mouse IgG secondary antibody labeled with HRP (Beijing ZSGB Biotechnology, ZDR-5307, 1:200) or the goat anti-rabbit IgG secondary antibody labeled with HRP (Beijing ZSGB Biotechnology, ZDR-5306, 1:200) was added and incubated at 37°C for 60 min. The slices were treated with 3,3′-diaminobenzidine tetrahydrochloride (DAB) to observe the results. The sections were counterstained with hematoxylin, dehydrated, and observed under an Olympus microscope. The sequential sections from all the collected tissues and organs were directly incubated with the goat anti-mouse IgG or goat anti-rabbit IgG secondary antibody labeled with HRP and prepared as the omission control for viral antigen staining. The sequential sections from these tissues were maintained in a recombinant anti-mouse IgG antibody [RM104] (Abcam, ab190475, 1:1000) or a recombinant anti-rabbit IgG antibody [SP137] (Abcam, ab208334, 1:1000) as the negative control for testing the level of viral antigen.

### *In situ* Hybridization (ISH)

To examine SARS-CoV-2 genomic RNA in FFPE tissues, ISH was applied by the RNAscope^®^ 2.5 HD Reagent Kit-RED (Advanced Cell Diagnostic, ACD, Cat No. 322310) depending on the manufacturer’s instructions (Hecht et al., 2020; Liu et al., 2020). Briefly, ISH Probe-V-nCoV2019-S (Advanced Cell Diagnostic, ACD, Cat No. 848561, positive-sense RNA probe) (Genomic RNA fragment 21631-23303, RefSeq #NC_045512.2) was prepared and synthesized by ACD. With xylene, the tissue sections were deparaffinized, underwent a series of ethanol washes and peroxidase blocking, and then were heated in an antigen retrieval buffer provided by the kit and digested in the proteinase provided by the kit. Sections were incubated in ISH target probe at 40°C in a hybridization oven for 2 h. After rinsing, ISH signal was amplified by the pre-amplifier provided by the kit and amplifier conjugated to alkaline phosphatase and incubated with DAB for visualization at room temperature. Then, the sections were stained with hematoxylin, air-dried, mounted, and stored at 4°C for observation and analysis.

### Statistical Analysis

The experimental data were analyzed with GraphPad Prism 8.0 software (GraphPad Software Inc., San Diego, CA, United States).

## Results

### Clinical Features, Viral RNA Distribution, and Histopathological Changes in Lung

**FIGURE 1:**
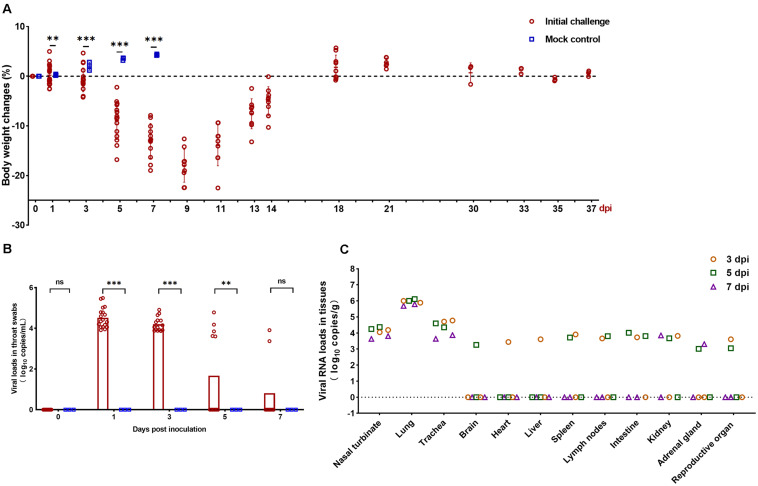
Clinical characteristics and viral load in the collected organs. **(A)** Body weight changes in Syrian hamsters after viral infection at 0, 1, 3, 5, 7, 9, 11, 13, 14, 18, 21, 30, 33, 35, and 37 days post inoculation (dpi). The mock-infected hamsters were examined at 0, 1, 3, 5, and 7 dpi. **(B)** Viral RNA (log10 RNA copies/mL) by qRT-PCR assay detected in the throat swabs at 0, 1, 3, 5, and 7 dpi. **(C)** Viral RNA (log10 RNA copies/g) by qRT-PCR assay detected in the respiratory tract tissues and extrapulmonary organ tissues of SARS-CoV-2-challenged hamsters at 3, 5, and 7 dpi. Statistical significance in **(A,B)** was calculated between infected and mock-infected animals within each group. The data shown are the mean ± SEM. Statistical significance between groups was calculated by *Student’s t-test* (^*ns*^*P* > 0.05, ^∗∗^*P* < 0.01, ^∗∗∗^*P* < 0.001).

SARS-CoV-2-infected but not mock-infected animals exhibited progressively body weight loss from 1 to 9 dpi. The infected animals exhibited severe weight loss at 5 days dpi (8.91%), which peaked at 9 dpi (18.02%), then gradually regained their weight by 14 dpi (5.04%). In contrast, the body weight of mock-infected hamsters gradually increased by 7 dpi (Figure 1A). The viral load was detected from throat swabs and peaked at 1 dpi (3.96 log_10_ RNA copies/mL) (Figure 1B). Next, viral RNA was detected by qRT-PCR in the respiratory tract (nasal turbinate, lung, and trachea) and extrapulmonary organs (brain, heart, liver, spleen, lymph nodes, intestine, kidney, adrenal gland, and reproductive organs) to investigate the dissemination of SARS-CoV-2 in hamsters. Infection of hamsters with SARS-CoV-2 resulted in a high mean viral RNA load in the nasal turbinate (4.05 log_10_ RNA copies/g), lung (5.91 log_10_ RNA copies/g), and trachea (4.33 log_10_ RNA copies/g) at 3–7 dpi (Figure 1C). For extrapulmonary organs, viral RNA was detectable in different organs at different days, the detectable rate progressively decreased from 3 to 7 dpi (Figure 1C).

**FIGURE 2:**
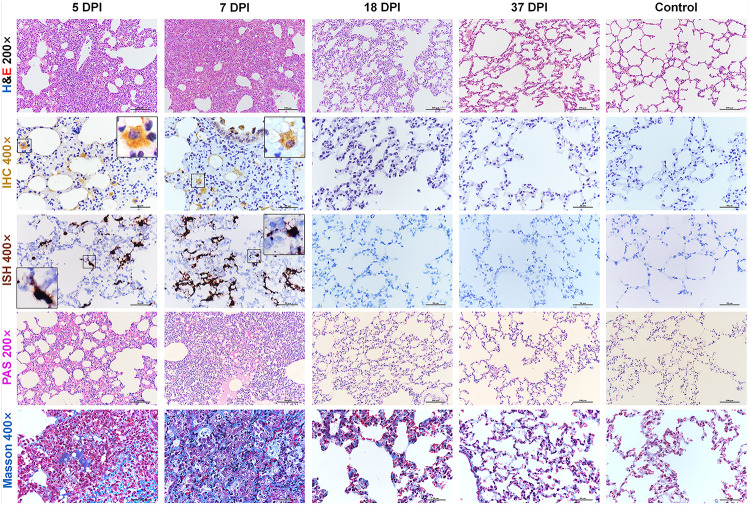
Histopathological changes of the lung at 5, 7, 18, 37 dpi. The viral pathology in the lungs was observed by hematoxylin and eosin (H&E) staining. Viral S protein was examined by immunohistochemistry (IHC). Viral RNA was detected by *in situ* hybridization (ISH). The black frames in the corners are a magnification of the region in the respective panel of IHC and ISH. Exudation was tested by periodic acid-Schiff (PAS) staining. Collagenous fibers were stained by modified Masson staining. Data are representative of three independent experiments.

We further examined the histopathological changes in the respiratory organs along with the period of disease. Consistent with previous reports (Imai et al., 2020; Sia et al., 2020), after 3 dpi, hamsters infected with SARS-CoV-2 developed moderate to severe lung lesions. At 5 and 7 dpi, the lesions progressed into multifocal and coalescent lesions throughout the lung lobes. Diffuse alveolar destruction, marked infiltration of inflammatory cells including macrophages, lymphocytes, and neutrophils, in the alveolar septum, cell debris filling bronchiolar lumina, and alveolar collapse with hemorrhage were observed. Viral S protein was abundantly expressed in bronchiolar epithelial cells, alveolar epithelial cells, and alveolar macrophages. Selected IHC results were further confirmed by ISH. Robust viral RNA expression was detected by RNAscope^®^ probe in the lungs at 5 and 7 dpi (Supplementary Figures S1A,B). No detectable viral antigen or RNA was present in the lungs collected at 18 or 37 dpi or from the mock control hamsters. Next, periodic acid-Schiff (PAS)-positive exudate could be observed in the alveolar lumina, alveolar septum and bronchiolar lumina at 5 and 7 dpi but not at 18 or 37 dpi. A small amount of collagen could be observed focally in the thickened alveolar interstitium by modified Masson’s trichrome staining at 5 dpi, and the amount of collagen was increased and more scattered at 7 dpi. Compared with the control mice, the infected hamsters showed a slight increase in the amount of collagen at 18 dpi. No substantial difference could be observed in the lungs between 37 dpi hamsters and control hamsters (Figure 2).

### SARS-CoV-2 Infects Multiple Extrapulmonary Organs in Hamsters Including Urogenital Organs and Adrenal Gland

At 5 and 7 dpi, all the infected hamsters displayed severe systemic inflammatory responses that led to severe interstitial pneumonia and different degrees of multiple organs lesions by H&E observation. All the collected tissues and organs were examined for both viral antigen (IHC) and RNA (ISH). The mainly results detected by qRT-PCR, IHC, and ISH were summarized in Table 1.

**TABLE 1:** The infectious multi-organs in hamsters with COVID-19.

Organ system	Days post inoculation														
	3			5			7			18			37		
	Detection methods														
	qPCR	IHC	ISH	qPCR	IHC	ISH	qPCR	IHC	ISH	qPCR	IHC	ISH	qPCR	IHC	ISH
Respiratory system															
Nasopharynges	+	+	+	+	+	+	+	+	+	–	–	–	-	-	-
Trachea	+	+	+	+	+	+	+	+	+	–	–	–	-	-	-
Lungs	+	+	+	+	+	+	+	+	+	–	–	–	-	-	-
Immune system															
Spleen	+	+	+	+	+	+	–	+	+	–	–	–	–	–	–
Lymph nodes	+	+	+	+	+	+	–	+	+	–	–	–	–	–	–
Digestive system															
Liver	+	±	±	–	–	–	–	–	–	–	–	–	–	–	–
Small intestine	+	+	+	+	+	+	–	–	–	–	–	–	–	–	–
Colon	+	+	+	+	+	+	–	–	–	–	–	–	–	–	–
Gallbladder	ND	–	–	ND	±	±	ND	–	–	ND	–	–	ND	–	–
Pancreas	ND	–	–	ND	±	±	ND	–	–	ND	–	–	ND	–	–
Urinary and genital systems															
Kidneys	+	+	+	+	+	+	+	+	+	–	–	–	–	–	–
Ovary	–	–	–	+	+	+	–	–	–	–	–	–	–	–	–
Testis	+	–	–	–	–	–	–	+	+	–	–	–	–	–	–
Vesicular gland	ND	–	–	ND	–	–	ND	+	+	ND	–	–	ND	–	–
Prostate	ND	–	–	ND	–	–	ND	+	+	ND	–	–	ND	–	–
Cardiovascular system															
Heart	+	–	–	–	–	–	–	–	–	–	–	–	–	–	–
Endocrine system															
Adrenal glands	–	–	–	+	+	+	+	+	+	–	–	–	–	–	–
Nervous system															
Cerebrum	–	–	–	+	±	±	–	–	–	–	–	–	–	–	–
Cerebellum	–	–	–	+	–	–	–	–	–	–	–	–	–	–	–

*PCR, polymerase chain reaction; IHC, immunohistochemistry; ISH, *in situ* hybridization; ND, no detection; +, positive result; –, negative result; ±, positive result to be confirmed.*

**FIGURE 3:**
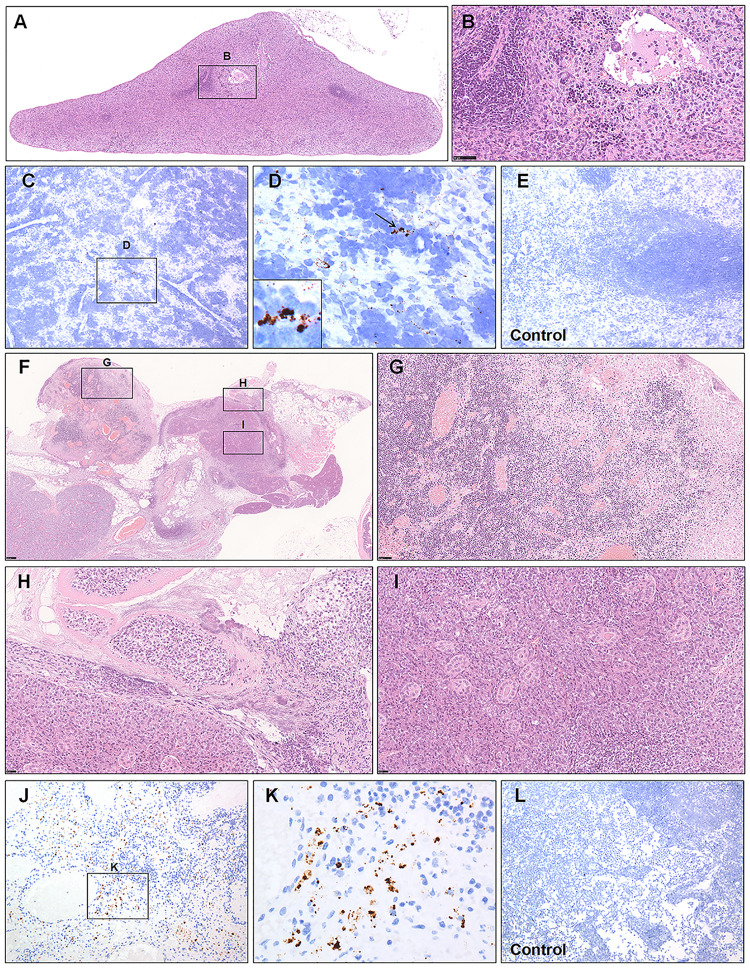
Observation and examination of the spleen and lymph nodes. **(A,B)** White and red pulp depletion with a reduction in the follicle number and size in infected hamsters at 7 dpi. **(C–E)** Low viral RNA expression was detected by ISH in the splenic marginal zone and red pulp compared with the control group at 7 dpi. The black frames in the corner of **(D)** is the magnification of the arrowed region. **(F–I)** The germinal center of the affected mandibular lymph node had disappeared and been displaced by massive necrotic areas at 5 dpi. **(J–L)** Some viral RNA expression was detected by ISH in the parenchyma of the medulla compared with the mock control group at 5 dpi. Sequential sections were stained by H&E and subjected to ISH. Data are representative of three independent experiments.

The significance of secondary lymphoid organs containing the spleen and lymph nodes (LNs), for resisting against infection is well defined. Tissue-resident macrophages placed in the splenic marginal zone are among the first cell types to bring up against invading pathogens (Aichele et al., 2003; Feng et al., 2020). Likewise, the resident macrophages of the subcapsular sinus and hilar LNs have been suggested to function as a protective role against viral infections by taking viral particles (Junt et al., 2007). Previous reports have demonstrated that lymphocytopenia is prevalent in patients with COVID-19 (Feng et al., 2020; Xu et al., 2020). Here, we observed white and red pulp depletion with reductions in follicle number and size in infected hamsters at 5 and 7 dpi (Figure 3A). Marked phagocytosis of debris or affected cells with swelling, degeneration, or necrosis was present around white pulp. Inflammatory cells and megakaryocytes were observed in the splenic vein (Figure 3B). A small amount of viral RNA was detected by ISH in the MZ and red pulp compared with that from the mock control group (Figures 3C–E). For one hamster at 5 dpi, the affected mandibular LN had been destroyed; the germinal center had disappeared and been displaced by massive necrotic areas (Figure 3F). The retropharyngeal LNs draining the nasal cavity, with acute rhinitis (Supplementary Figures S1C–E), may develop acute lymphadenitis. The LNs were markedly congested, and the sinuses were filled with blood. The sinuses and the parenchyma of the cortex and medulla showed coalescing foci of neutrophilic inflammation, necrosis, and fibrin deposition (Figure 3G). Some veins were filled with swollen and degenerative inflammatory cells or debris (Figure 3H). The affected submandibular glands were also surrounded by inflammatory cells and necrosis, and some debris or exudate filled the lumen of the glands (Figure 3I). Some viral RNA expression was detected by ISH in the parenchyma of the medulla compared with that from the mock control group (Figures 3J–L).

**FIGURE 4:**
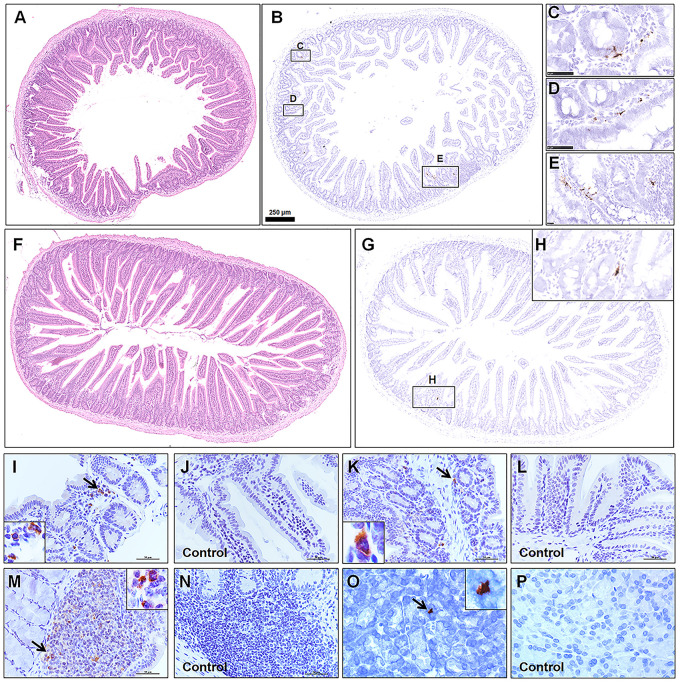
Observation and examination of the alimentary system at 5 dpi. **(A–E)** Observation of the duodenum by H&E and examination by ISH. **(F–H)** Observation of the jejunum by H&E and examination by ISH. **(I–P)** The ileum, colon, Peyer’s lymph nodes, and pancreas tissues from the infected group and mock control group were examined by IHC. The black frames in the corners are a magnification of the arrowed region in the respective panel. Sequential sections were stained by H&E and subjected to ISH and IHC. Data are representative of three independent experiments.

Consistent with previous report, viral antigen was detected in the alimentary tract. Furthermore, different segments of the alimentary system, including the duodenum (Figure 4A, HE; Figures 4B–E, ISH), jejunum (Figure 4F, HE; Figures 4G,H, ISH), The ileum (Figures 4I,J), colon (Figures 4K,L), Peyer’s LNs (Figures 4M,N), and pancreas tissues (Figures 4O,P) from the infected group and mock control group were examined by IHC. Most of the intestinal mucosal epithelial cells were intact. Focal mononuclear or neutrophilic cell infiltration was observed in the lamina propria. Although there were no marked lesions present in the alimentary system, rare weak viral RNA and antigen expression was detected by ISH and IHC, mainly in the lamina propria or submucosa, in a patchy distribution. Some viral antigen was also observed in Peyer’s LNs in the ileum, implying that the virus might spread from the initial entry through gut-associated lymphoid tissues.

**FIGURE 5:**
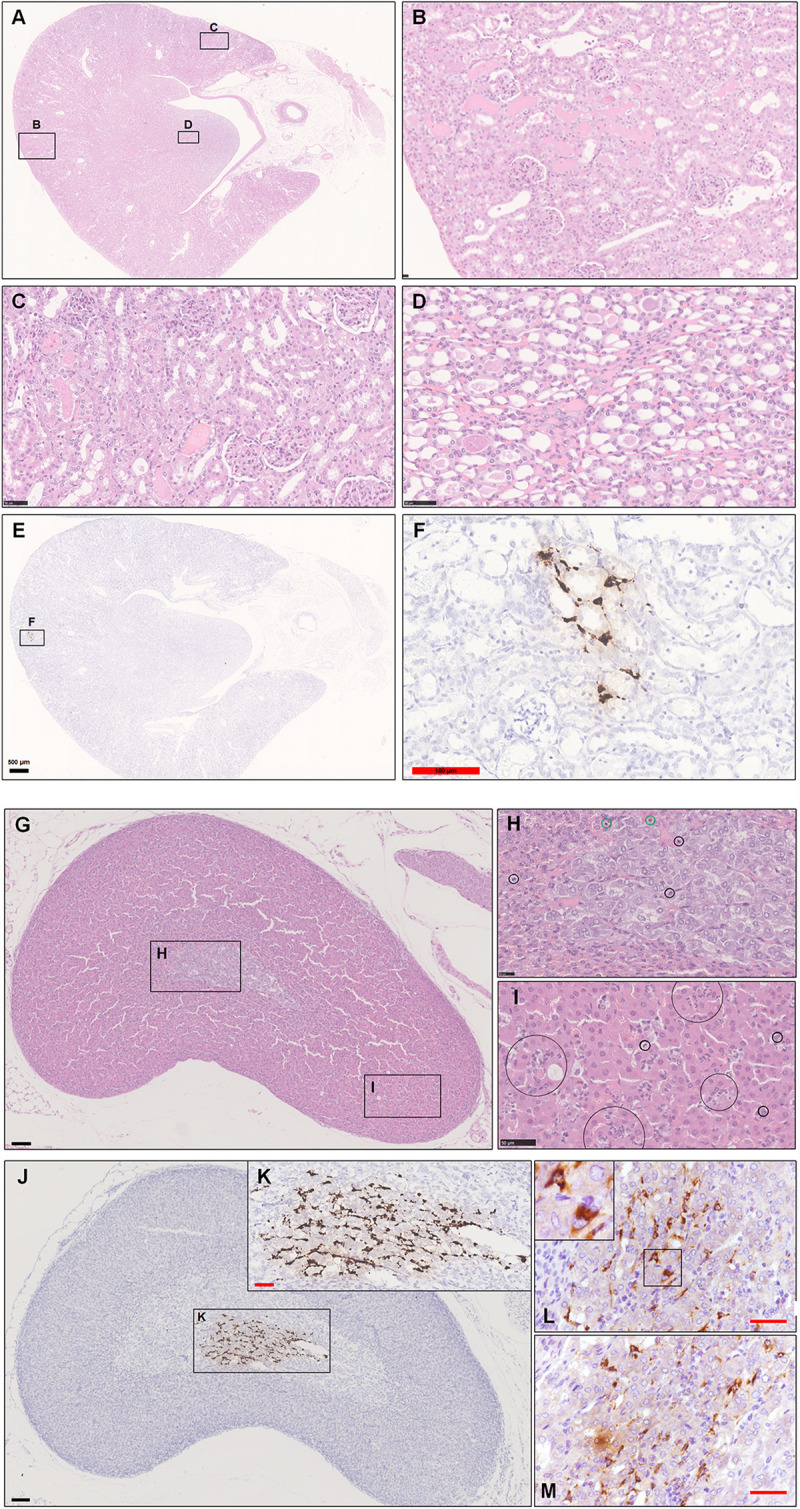
Observation and examination of the kidney and adrenal glands at 5 dpi. **(A–D)** The kidney lesions were characterized primarily by coagulation necrosis with occasional detachment of the epithelium from the tubular basement membrane. Some proteinaceous debris was present in the dilated proximal and distal tubules or collecting tubules. **(E,F)** Low viral RNA expression was also detected by ISH in the renal tubules. **(G–I)** Scattered inflammatory cells, mainly including neutrophilic cells (black circle in **H,I**) and lymphocytes (green circle in **H**), in the adrenal cortex and medulla. **(J,K)** Robust viral RNA expression was detected by ISH in the adrenal medulla. **(L,M)** Abundant viral S protein expression was detected by IHC in the cytoplasm. The black frames in the corner of L is the magnification of the framed region. Sequential sections were stained by H&E and subjected to ISH, IHC, and IF. Data are representative of three independent experiments.

For kidney, we found that some glomeruli were atrophied, and renal tubules were damaged, characterized by acute tubular necrosis, mainly in the renal cortex (Figures 5A,B). This lesion was characterized primarily by coagulation necrosis with occasional detachment of the epithelium from the tubular basement membrane, nuclear pyknosis, karyorrhexis, and karyolysis. Some proteinaceous debris was present in the dilated proximal or distal tubules. Infiltration by inflammatory cells, including monocytes, lymphocytes and neutrophilic cells, was observed in the renal interstitium (Figure 5C). The lumen of some of the collecting tubules was also filled with proteinaceous debris (Figure 5D). Consistently, low viral RNA expression was also detected by ISH in the renal tubules (Figures 5E,F).

During this research, we also collected the adrenal gland, which was named for its position just craniomedial to the kidney. The adrenal cortex is derived from mesodermal origin, and adrenocortical cells produce corticosteroids (steroid hormones) from cholesterol, whereas the adrenal medulla has a neural crest ectoderm origin, and its cells synthesize catecholamines from tyrosine. The adrenal medulla is enclosed by the adrenal cortex, so medullary cells are incubated in cortisol-rich blood. This close anatomic combination between the adrenal cortex and medulla is crucial because the phenylethanolamine-*N*-methyltransferase that converts norepinephrine to epinephrine is corticosteroid hormone-dependent (Zachary and McGavin, 2012).

First, we observed scattered inflammatory cells, mainly including lymphocytes and neutrophilic cells, in the adrenal cortex and medulla in one infected hamster at 5 dpi. Some chromaffin cells were swollen or necrotic. The adrenal medulla appeared to have become atrophied (Figures 5G–I). Next, we examined this adrenal tissue by ISH and IHC. To our surprise, there was robust viral RNA expression in a dense distribution (Figures 5J,K). The viral protein was mainly expressed in the cytoplasm of some spindle or triangle cells, according to staining for SARS-CoV-2 S protein (Figures 5L,M). At 7 dpi, all adrenal glands from the three hamsters were tested by IHC and ISH. Two-thirds of them exhibited weak viral RNA expression in the adrenal medulla.

**FIGURE 6:**
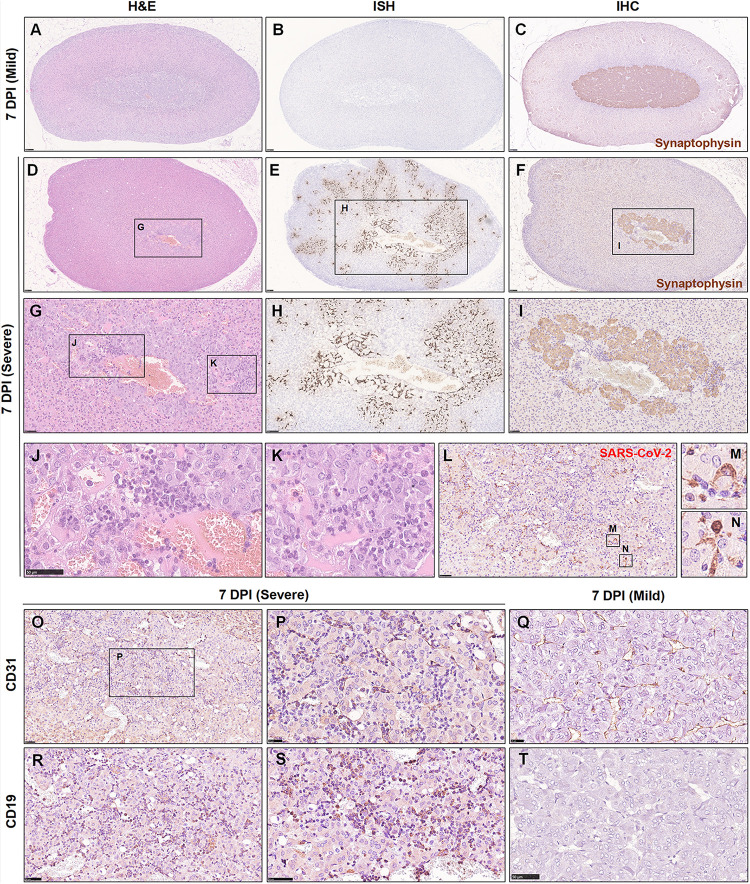
The adrenal gland was severely affected, and SARS-CoV-2 was detected throughout the adrenal gland at 7 dpi. **(A–C)** Mild adrenal gland changes accompanied by few inflammatory foci were observed in one infected hamster at 7 dpi. **(D,G,J,K)** Multifocal inflammatory cells, mainly including neutrophilic cells and lymphocytes, were scattered in the adrenal cortex and medulla. **(E,H)** Robust viral RNA expression was detected by ISH in the adrenal cortex and medulla. **(F,I)** The adrenal medulla was atrophied, as confirmed by synaptophysin protein staining. **(L–N)** The expression of viral S protein was further confirmed by IHC, mainly in the cellular cytoplasm. **(O–Q)** Abundant vascular endothelial cells were stained for a biomarker, CD31. **(R–T)** A mass of CD19-positive cells was stained, confirming the aggregation of B lymphocytes. Sequential sections were stained by H&E and subjected to ISH and IHC. Data are representative of three independent experiments.

The above findings brought this organ to our attention. Initially, we collected and embedded the adrenal glands together with the kidney; this gland was so small that it could not be well presented in sections together with other larger organs. Therefore, an independent set of 15 adrenal gland samples at 7 dpi was collected. All tissues were embedded separately and observed by H&E staining and examined by IHC and ISH (Table 2). Remarkably, in 15/18 hamsters, focal to multifocal inflammation was observed in the adrenal cortex and medulla. Among the 15 hamsters, 13 of them exhibited viral RNA as detected by ISH and viral antigen as detected by IHC. Six of the detectable adrenal glands tested positive for viral RNA not only in the adrenal medulla but also throughout the adrenal cortex. As the adrenal medulla is derived from neural crest ectoderm, one marker protein of the adrenal medulla, synaptophysin, which is expressed in vesicles of various neuroendocrine cells of both neuronal and epithelial phenotypes, was used to examine the viral distribution in this case. Compared with the adrenal gland, which displayed mild or rare pathological changes (Figures 6A–C), the adrenal gland, which presented severe inflammation throughout the organ, also exhibited adrenal medulla atrophy (smaller area of medulla stained by synaptophysin in Figure 6F) and swelling, along with degenerative or necrotic adrenocortical cells and chromaffin cells (Figures 6D–I). In the center of the adrenal medulla, the small vessels seemed to be increased in number, and there were masses of inflammatory cells, including neutrophilic cells, lymphocytes, and mononuclear cells (Figures 6G,J–N). Next, staining for CD31, the biomarker of vascular endothelial cells, was performed to clearly observe the small vessels (Figures 6O,P) compared with those in animals with mild changes (Figure 6Q). Furthermore, CD19-positive cells were aggregated in the inflammatory foci (Figures 6R,S) compared with those in animals with mild changes (Figure 6T). In a word, adrenal gland is a target organ vulnerable to infection by SARS-CoV-2.

**TABLE 2:** Summary of lesions in adrenal glands induced by SARS-CoV-2 at 7 dpi.

Degree	H&E	ISH	IHC	Notes
Normal **(3/18)**	No substantial pathological change **(3/18)**	No detectable viral RNA **(3/18)**	No detectable viral S protein **(3/18)**	The ratings of the three hamsters were the same.
Mild **(2/18)** to moderate **(7/18)**	Focal **(2/18)** to multifocal **(7/18)** inflammation	Low viral RNA expression in the adrenal medulla **(7/18)**	Weak viral S protein expression in the adrenal medulla **(7/18)**	In 2/18 hamsters, there was focal inflammation without detectable viral RNA or antigen.
Severe **(6/18)**	Diffuse inflammation **(6/18)**	Robust viral RNA expression in the adrenal cortex and medulla **(6/18)**	Viral S protein detected mainly in the adrenal medulla **(6/18)**	The ratings of the three hamsters were the same.

**FIGURE 7:**
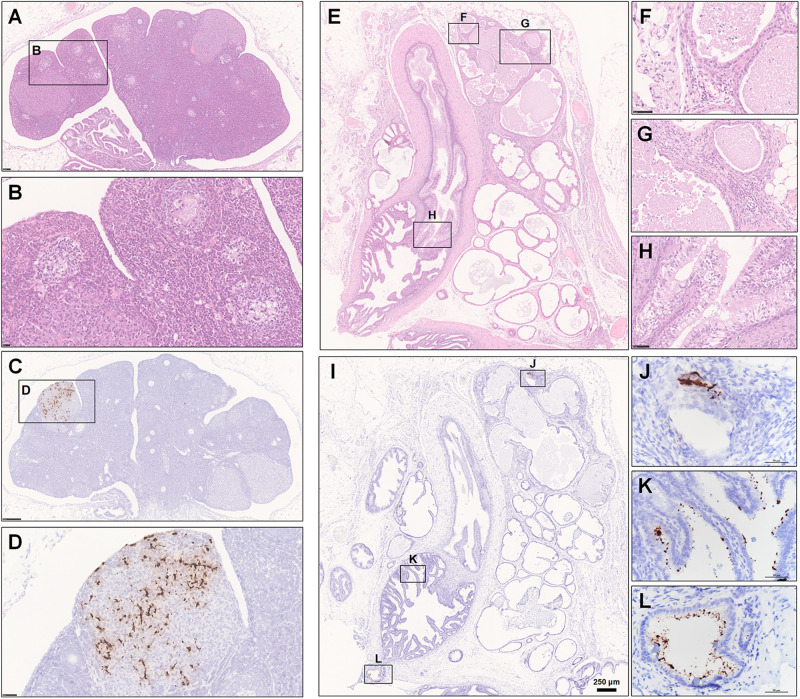
Observation and examination of the reproductive system. **(A–H)** The female reproductive system at 5 dpi. **(A,B)** Oophoritis was present, characterized by focal necrosis and the presence of mononuclear cells and lymphocytes in the stromal connective tissue. No mature follicles were found. **(C,D)** Robust viral RNA expression was detected in the area of the corpus luteum. **(E–L)** The male accessory sexual gland at 7 dpi. **(E–H)** Prostatitis and inflammation of the vesicular gland. **(I–L)** Robust viral RNA expression was detected in a patchy distribution in the interstitium of the prostate and the epithelium of the vesicular glands. Sequential sections were stained by HE and subjected to ISH. Data were representative of three independent experiments.

The female and male reproductive systems were both observed by H&E staining and examined by ISH and IHC. Normally, the cortex of the ovary contains different stages of developing follicles. When the follicle ruptures, ovulation occurs, releasing the ovum and allowing the space to be swarming with blood and then with luteal cells to form the corpus hemorrhagicum and corpus luteum, respectively. However, in one infected hamster at 5 dpi, oophoritis was present, characterized by focal necrosis and the presence of mononuclear cells and lymphocytes in the stromal connective tissue. Some follicles even displayed necrosis, including granulosa cells. No mature follicles were found (Figures 7A,B). Notably, robust viral RNA expression was detected in the area of the corpus luteum (Figures 7C,D). Regarding the male reproductive system, no substantial pathological changes were observed in the testis, while rare weak expression of viral RNA was detected by ISH in Sertoli cells (Supplementary Figure S1F); this anti-inflammatory result is perhaps due to the blood-testis barrier, which results in an altered immunologic environment in the testis. There were some lesions in the prostate and the vesicular gland (Figure 7E). Prostatitis (Figures 7F,G) and inflammation of the vesicular gland (Figure 7H) were present. The glands and interstitium contained large numbers of inflammatory cells, including lymphocytes, mononuclear cells, and neutrophils. Some debris was desquamated into the lumen of the prostate. The epithelium of the vesicular gland was vacuolated, swollen and degenerated. Remarkably, robust viral RNA expression (Figure 7I) was detected in a patchy distribution in the interstitium of the prostate (Figure 7J and Supplementary Figures S1G,H) and the epithelium of the vesicular gland (Figures 7K,L). There were no detectable viral RNA could by examined in the collected tissues from the normal control hamsters by ISH (Supplementary Figures S1I–K).

### Convalescent Pathology of Infected Hamsters

**FIGURE 8:**
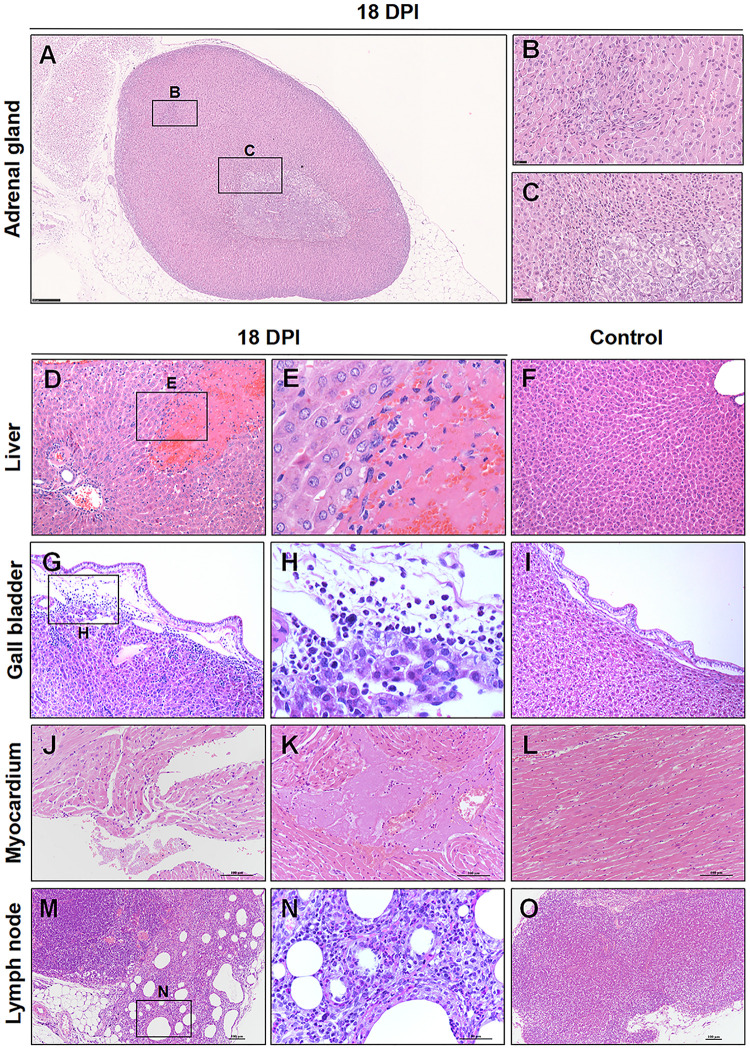
Observation and examination of infected hamsters at 18 dpi. **(A–C)** Regeneration was present in the adrenal glands at 18 dpi. **(D–F)** Compared with the normal control liver, there was focal hepatocellular necrosis in the liver. **(G–I)** Compared with the normal control gallbladder, there were inflammatory cells in the submucosa of the gallbladder. **(J–L)** Compared with the normal control myocardium, the heart showed mild focal myocardial degeneration and inflammation. **(M–O)** Compared with the normal control lymph node, chronic lymphadenitis accompanied by fibrosis was present in one hamster at 18 dpi.

At 18 dpi, there were a few pathological changes can be observed in the hamsters. There were no viral RNA or viral S protein can be detected at 18 and 37 dpi. Notably, there were regeneration present in the adrenal glands, which suggesting that if the hamster passed the crisis, the damaged adrenal glands might be recovery slowly (Figures 8A–C). Additionally, there were a random distribution of hepatocellular necrosis in the liver with rare inflammation, which similar like other viral infection in the liver, such as herpesvirus infection in many species (Zachary and McGavin, 2012) (Figures 8D,E), compared with the normal control liver (Figure 8F). Cholecystitis, inflammation of the gallbladder, was present in the hamster. The mucosa and submucosa of the gallbladder were edema. The inflammatory cells were mainly lymphocytes, neutrophils, and mononuclear cells in the submucosa (Figures 8G,H), compared with the normal control gallbladder (Figure 8I). The heart showed mild focal myocardial degeneration and inflammatory (Figures 8J,K), compared with the normal control myocardium (Figure 8L). Additionally, there was chronic lymphadenitis accompanied with fibrosis present in one hamster (Figures 8M,N), compared with the normal control LN (Figure 8O).

## Discussion

Albeit extrapulmonary infection perhaps less common in mild or subclinical disease and not easy to examine for clinical patients, it is unknown whether active extrapulmonary infection can be present in a patient without concurrent infection of respiratory system. Our findings of systemically multi-organs involvement of SARS-CoV-2 provide significant data to support the clinical symptoms found in patients with COVID-19 during periods of disease and convalescence.

Recently, a report of clinical trials that measured the impact of corticosteroids compared with standard care or a placebo on patients with COVID-19 displayed statistically significant difference. Corticosteroids are both affordable and easily available medicine with anti-inflammatory outcomes that trials have recommended could resist dangerous COVID-19 complications (McIntosh, 2020). Of 628 patients who received the treatment of steroid, the analysis, conducted by the WHO and posted in the Journal of the American Medical Association on September 2,2020, demonstrated that approximately thirty-three percent passed away over a 28-day observation period. Approximately 41% of 1,025 patients who received standard care or a placebo died during the same period. Using meta-analyses, statistical modulations, reflected an absolute mortality risk reduction of approximately 30% when corticosteroids were applied (Zha et al., 2020). It is well known that the adrenal gland is responsible for producing mineralocorticoids, aldosterone, and glucocorticoids by adrenal medullary cells producing the catecholamine hormones norepinephrine and epinephrine from tyrosine. Our findings demonstrate serious injury to the adrenal gland in 6/18 hamsters at 7 dpi. In severe cases, diffuse inflammatory cells mainly included lymphocytes and neutrophilic cells in the adrenal cortex and medulla. A mass of viral RNA was detected at 5 dpi in the adrenal medulla and then spread throughout the adrenal gland at 7 dpi. Functionally, the adrenal medulla and the cortex are two relatively independent organs with connected anatomy, but they share the same blood supply, suggesting a potential pathway for the spread of pathogens. Our research found serious pathological changes in approximately 33% of the adrenal glands collected from infected hamsters at 7 dpi. These hamsters might lose the ability to produce mineralocorticoids, aldosterone, glucocorticoids, catecholamine hormones, norepinephrine and epinephrine by themselves. Consistently, the adrenal glands of patients with clinically severe COVID-19 may have been affected, accompanied by dysfunctional hormone production and disorder of the endocrine system. Additionally, not all adrenal glands from hamsters were detectable affected by SARS-CoV-2, which is consistent with the recommendations of the WHO emphasizing that corticosteroid treatment, while apparently beneficial for patients suffering from severe cases of COVID-19, does not appear to have an effect on patients with non-critical cases of the disease in the same way. Under normal conditions, the body has different degrees of physiological compensation, regeneration and repair activity during stress or injury. SARS-CoV-2 not only induces severe respiratory system lesions but also damages multiple extrapulmonary organs (Bradley et al., 2020). In severely affected patients who also had significant comorbidities, the adrenal gland is much more vulnerable.

The adrenal cortex includes the zona glomerulosa, fasciculata, and reticularis. The zona glomerulosa produces mineralocorticoids, primarily aldosterone. Aldosterone regulates blood pressure and extracellular fluid volume by operating at the distal and collecting tubules of the kidney to facilitate sodium retention and potassium excretion. The zona fasciculata, located in the middle, is the largest layer of the cortex. Its cells synthesize cortisol and other glucocorticoids; therefore, it responds to stimulation by adrenocorticotrophic hormone (ACTH) released into the systemic circulation by the adenohypophysis (anterior pituitary gland) (Zachary and McGavin, 2012). Glucocorticoids have different effects on many tissues and organs throughout the body, but generally, they are inclined to enhance glucose production, reduce lipogenesis, restrain the immune response, and suppress inflammation and its repair by fibroplasia (Hammer and McPhee, 2014). Adrenal medullary cells synthesize the catecholamine hormones norepinephrine, epinephrine, and dopamine. Catecholamines help to regulate metabolism, cardiac and smooth muscle contractility, and neurotransmission. Physiologic stimuli affect medullary secretion through the nervous system. Catecholamine secretion is low in the basal state and is reduced even further during sleep. In emergency situations, there is increased adrenal catecholamine secretion as part of a generalized sympathetic discharge that serves to prepare the individual for stress. Hypoglycemia and certain drugs are also potent stimuli for catecholamine secretion. Patients who have combined deficiencies of the adrenal cortex and medulla will suffer an increased risk for COVID-19. These functions of the adrenal gland, which might normally be compensatory, are particularly important after viral infection but are so vulnerable and susceptible that they may aggravate disease progression and even lead to death.

Previous reports have mainly focused on the respiratory system. Among non-respiratory tract tissues, only intestinal tissue demonstrated viral antigen expression. Here, we found damage in multiple extrapulmonary organs. The mass of viral RNA expression in the corpus luteum of the ovary, vesicular gland and prostate suggests that SARS-CoV-2 might affect human and animal reproduction and development, which requires further research. Low viral RNA expression was also detected in tubular cells accompanied by acute tubular cell necrosis. In fact, one of the first events in renal tubular cell damage is changed ion transport at the luminal surface (uptake). This course leads to reduced sodium absorption and enhanced sodium ions in the lumina of the distal tubules, which activate the renin-angiotensin mechanism, inducing vasoconstriction and reduced blood flow, in turn resulting in ischemia and tubular cell damage. In severe cases, the renin-angiotensin mechanism related to the adrenal cortex also aggravates kidney injury.

The lesions in multiple organs induced by SARS-CoV-2 in hamsters need to be further research whether there exist disorder of their function during disease and even after recovery. Our findings illustrate systemic histological observations and the viral RNA and viral S protein distribution in infected hamsters, contributing to the pathophysiologic characterization of SARS-CoV-2-induced systemic disease and hopefully providing evidence supporting the development of effective treatment strategies for clinical patients.

## Data Availability Statement

The complete genome for this SARS-CoV-2 was submitted to NCBI (SARS-CoV-2/WH-09/human/2020/CHN, accession code MT093631.2). All raw data are available from the corresponding author upon reasonable request. Source data are provided with this paper as a source data file.

## Ethics Statement

The animal study was reviewed and approved by the Institutional Animal Care and Use Committee of the Institute of Laboratory Animal Science, Peking Union Medical College.

## Author Contributions

CQ: conceptualization, resources, supervision, and writing – review and editing. CQ, ZS, LB, and PY: methodology. CQ, ZS, LB, PY, WD, SG, JL, JW, BZ, FQ, JX, QW, ML, WZ, and YH: investigation. ZS and JX: writing – original draft. CQ, ZS, and LB: funding acquisition. All authors contributed to the article and approved the submitted version.

## Conflict of Interest

The authors declare that the research was conducted in the absence of any commercial or financial relationships that could be construed as a potential conflict of interest.

## Supplementary Material

Supplementary Figure 1 Observation and examination of lung, nasal cavity, testis, and accessory sexual gland at 7 dpi. **(A,B)** There were amounts of viral Rna in the coalescent lesions throughout the lung lobes by Ish. **(C–E)** The epithelium of the nasal cavity were tested a mass of viral Rna by Ish. **(F)** Rare weak expression of viral Rna was detected by Ish in the Sertoli cell. The black frames in the corner is a magnification of the arrowed region in **(F)**. **(G,H)** Robust expression of viral Rna were detected in a patchy distribution in the lumen and interstitium of the prostate. The black frames in the corner is a magnification of the arrowed region in **(H)**. **(I–K)** The mock control tissues from the testis, prostate, and nasal cavity were examined by Ish. Sequential sections were stained by He and subjected to Ish. Data were representative of three independent experiments.

## References

[B1] Aichele P. Zinke J. Grode L. Schwendener R. A. Kaufmann S. H. Seiler P. (2003). Macrophages of the splenic marginal zone are essential for trapping of blood-borne particulate antigen but dispensable for induction of specific T cell responses. *J. Immunol*. 171 1148–1155. 12874200 10.4049/jimmunol.171.3.1148

[B2] Bao L. Deng W. Huang B. Gao H. Liu J. Ren L. (2020a). The pathogenicity of SARS-CoV-2 in hACE2 transgenic mice. *Nature* 583 830–833. 10.1038/s41586-020-2312-y 32380511

[B3] Bao L. Gao H. Deng W. Lv Q. Yu H. Liu M. (2020b). Transmission of SARS-CoV-2 via close contact and respiratory droplets among hACE2 mice. *J. Infect. Dis*. 222 551–555. 32444876 10.1093/infdis/jiaa281 PMC7313959

[B4] Boudewijns R. Thibaut H. J. Kaptein S. J. Li R. Vergote V. Seldeslachts L. (2020). STAT2 signaling as double-edged sword restricting viral dissemination but driving severe pneumonia in SARS-CoV-2 infected hamsters. *BioRxiv* [Preprint]. 10.1101/2020.04.23.056838 PMC7672082 33203860

[B5] Bradley B. T. Maioli H. Johnston R. Chaudhry I. Fink S. L. Xu H. (2020). Histopathology and ultrastructural findings of fatal COVID-19 infections in Washington State: a case series. *Lancet* 396 320–332. 10.1016/S0140-6736(20)31305-2 32682491 PMC7365650

[B6] Carfì A. Bernabei R. Landi F. (2020). Persistent symptoms in patients after acute COVID-19. *Jama* 324 603–605. 10.1001/jama.2020.12603 32644129 PMC7349096

[B7] Chan J. F. Zhang A. J. Yuan S. Poon V. K. Chan C. C. Lee A. C. (2020). Simulation of the clinical and pathological manifestations of Coronavirus Disease 2019 (COVID-19) in golden Syrian hamster model: implications for disease pathogenesis and transmissibility. *Clin. Infect. Dis.* 26:ciaa325. 10.1093/cid/ciaa325 32215622 PMC7184405

[B8] de Wit E. Prescott J. Baseler L. Bushmaker T. Thomas T. Lackemeyer M. G. (2013). The middle East respiratory syndrome coronavirus (MERS-CoV) does not replicate in Syrian hamsters. *PLoS One* 8:e69127. 10.1371/journal.pone.0069127 23844250 PMC3699510

[B9] Deng W. Bao L. Gao H. Xiang Z. Qu Y. Song Z. (2020a). Ocular conjunctival inoculation of SARS-CoV-2 can cause mild COVID-19 in rhesus macaques. *Nat. Commun.* 11:4400. 10.1038/s41467-020-18149-6 32879306 PMC7467924

[B10] Deng W. Bao L. Liu J. Xiao C. Liu J. Xue J. (2020b). Primary exposure to SARS-CoV-2 protects against reinfection in rhesus macaques. *Science* 369 818–823. 10.1126/science.abc5343 32616673 PMC7402625

[B11] Feng Z. Diao B. Wang R. Wang G. Wang C. Tan Y. (2020). The novel severe acute respiratory syndrome coronavirus 2 (SARS-CoV-2) directly decimates human spleens and lymph nodes. *MedRxiv* [Preprint] 10.1101/2020.03.27.20045427

[B12] Gao Q. Bao L. Mao H. Wang L. Xu K. Yang M. (2020). Development of an inactivated vaccine candidate for SARS-CoV-2. *Science* 369 77–81. 10.1126/science.abc1932 32376603 PMC7202686

[B13] Hammer G. D. McPhee S. J. (2014). *Pathophysiology of Disease: An Introduction to Clinical Medicine 7/E*. New York, NY: McGraw-Hill Education.

[B14] Hecht J. L. Quade B. Deshpande V. Mino-Kenudson M. Ting D. T. Desai N. (2020). SARS-CoV-2 can infect the placenta and is not associated with specific placental histopathology: a series of 19 placentas from COVID-19-positive mothers. *Mod. Pathol.* 33 2092–2103. 10.1038/s41379-020-0639-4 32741970 PMC7395938

[B15] Imai M. Iwatsuki-Horimoto K. Hatta M. Loeber S. Halfmann P. J. Nakajima N. (2020). Syrian hamsters as a small animal model for SARS-CoV-2 infection and countermeasure development. *Proc. Natl. Acad. Sci. U.S.A.* 117 16587–16595. 32571934 10.1073/pnas.2009799117 PMC7368255

[B16] Junt T. Moseman E. A. Iannacone M. Massberg S. Lang P. A. Boes M. (2007). Subcapsular sinus macrophages in lymph nodes clear lymph-borne viruses and present them to antiviral B cells. *Nature* 450 110–114. 10.1038/nature06287 17934446

[B17] Liu J. Babka A. M. Kearney B. J. Radoshitzky S. R. Kuhn J. H. Zeng X. (2020). Molecular detection of SARS-CoV-2 in formalin fixed paraffin embedded specimens. *bioRxiv* [Preprint]. 10.1101/2020.04.21.042911 32379723 PMC7406253

[B18] Mao L. Jin H. Wang M. Hu Y. Chen S. He Q. (2020). Neurologic manifestations of hospitalized patients with coronavirus disease 2019 in Wuhan, China. *JAMA Neurol.* 77 683–690. 32275288 10.1001/jamaneurol.2020.1127 PMC7149362

[B19] McIntosh J. J. (2020). Corticosteroid guidance for pregnancy during COVID-19 pandemic. *Am. J. Perinatol.* 37 809–812. 10.1055/s-0040-1709684 32274772 PMC7356057

[B20] Roberts A. Vogel L. Guarner J. Hayes N. Murphy B. Zaki S. (2005). Severe acute respiratory syndrome coronavirus infection of golden Syrian hamsters. *J. Virol.* 79 503–511. 15596843 10.1128/JVI.79.1.503-511.2005 PMC538722

[B21] Sia S. F. Yan L. Chin A. W. H. Fung K. Choy K. Wong A. Y. L. (2020). Pathogenesis and transmission of SARS-CoV-2 in golden hamsters. *Nature* 583 834–838. 10.1038/s41586-020-2342-5 32408338 PMC7394720

[B22] Song Z. Xu Y. Bao L. Zhang L. Yu P. Qu Y. (2019). From SARS to MERS, thrusting coronaviruses into the spotlight. *Viruses* 11:59. 10.3390/v11010059 PMC6357155 30646565

[B23] Wang Y. Miao J. Chard L. Wang Z. (2019). Syrian hamster as an animal model for the study on infectious diseases. *Front. Immunol.* 10:2329. 10.3389/fimmu.2019.02329 PMC6781508 31632404

[B24] Xu Z. Shi L. Wang Y. Zhang J. Huang L. Zhang C. (2020). Pathological findings of COVID-19 associated with acute respiratory distress syndrome. *Lancet Respir. Med.* 8 420–422. 10.1016/S2213-2600(20)30076-X 32085846 PMC7164771

[B25] Yu P. Qi F. Xu Y. Li F. Liu P. Liu J. (2020). Age−related rhesus macaque models of COVID−19. *Anim. Models Exp. Med.* 3 93–97. 10.1002/ame2.12108 PMC7167234 32318665

[B26] Zachary J. F. McGavin M. D. (2012). *Pathologic Basis of Veterinary Disease5: Pathologic Basis of Veterinary Disease*. Amsterdam: Elsevier Health Sciences.

[B27] Zha L. Li S. Pan L. Tefsen B. Li Y. French N. (2020). Corticosteroid treatment of patients with coronavirus disease 2019 (COVID −19). *Med. J. Austr.* 212 416–420. 10.5694/mja2.50577 32266987 PMC7262211

