# Supplementary figures and images for: SARS-CoV-2 Causes a Systemically Multiple Organs Damages and Dissemination in Hamsters
Source: Front Microbiol. 2021 Jan 12;11:618891. doi: 10.3389/fmicb.2020.618891 (PMC7835519; doi:10.3389/fmicb.2020.618891)

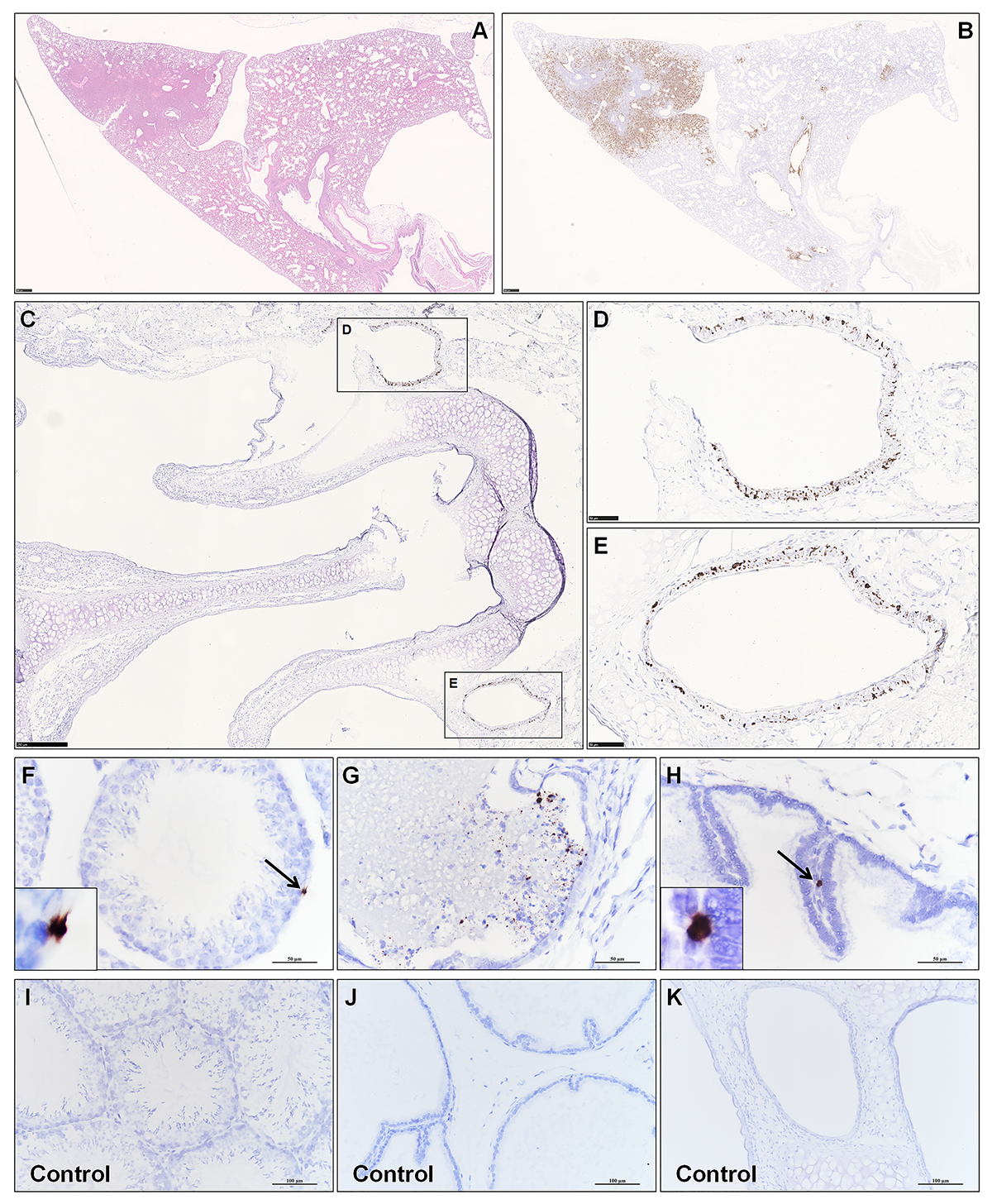

Supplement: Supplementary Figure 1 — Observation and examination of lung, nasal cavity, testis, and accessory sexual gland at 7 dpi. (A,B) There were amounts of viral Rna in the coalescent lesions throughout the lung lobes by Ish. (C–E) The epithelium of the nasal cavity were tested a mass of viral Rna by Ish. (F) Rare weak expression of viral Rna was detected by Ish in the Sertoli cell. The black frames in the corner is a magnification of the arrowed region in (F). (G,H) Robust expression of viral Rna were detected in a patchy distribution in the lumen and interstitium of the prostate. The black frames in the corner is a magnification of the arrowed region in (H). (I–K) The mock control tissues from the testis, prostate, and nasal cavity were examined by Ish. Sequential sections were stained by He and subjected to Ish. Data were representative of three independent experiments. [file Figure_1.TIF]
